# Advancing non-alcoholic fatty liver disease prediction: a comprehensive machine learning approach integrating SHAP interpretability and multi-cohort validation

**DOI:** 10.3389/fendo.2024.1450317

**Published:** 2024-10-08

**Authors:** Bo Yang, Huaguan Lu, Yinghui Ran

**Affiliations:** ^1^ Department of Gastroenterology and Hepatology, Guizhou Aerospace Hospital, Zunyi, China; ^2^ Technology Innovation Center, Hunan University of Chinese Medicine, Changsha, China; ^3^ Department of Gastroenterology, Affiliated Hospital of Zunyi Medical University, Zunyi, China

**Keywords:** non-alcoholic fatty liver disease, machine learning, SHAP interpretability, light gradient boosting machine, predictive model

## Abstract

**Introduction:**

Non-alcoholic fatty liver disease (NAFLD) represents a major global health challenge, often undiagnosed because of suboptimal screening tools. Advances in machine learning (ML) offer potential improvements in predictive diagnostics, leveraging complex clinical datasets.

**Methods:**

We utilized a comprehensive dataset from the Dryad database for model development and training and performed external validation using data from the National Health and Nutrition Examination Survey (NHANES) 2017–2020 cycles. Seven distinct ML models were developed and rigorously evaluated. Additionally, we employed the SHapley Additive exPlanations (SHAP) method to enhance the interpretability of the models, allowing for a detailed understanding of how each variable contributes to predictive outcomes.

**Results:**

A total of 14,913 participants were eligible for this study. Among the seven constructed models, the light gradient boosting machine achieved the highest performance, with an area under the receiver operating characteristic curve of 0.90 in the internal validation set and 0.81 in the external NHANES validation cohort. In detailed performance metrics, it maintained an accuracy of 87%, a sensitivity of 92.9%, and an F1 score of 0.92. Key predictive variables identified included alanine aminotransferase, gammaglutamyl transpeptidase, triglyceride glucose–waist circumference, metabolic score for insulin resistance, and HbA1c, which are strongly associated with metabolic dysfunctions integral to NAFLD progression.

**Conclusions:**

The integration of ML with SHAP interpretability provides a robust predictive tool for NAFLD, enhancing the early identification and potential management of the disease. The model’s high accuracy and generalizability across diverse populations highlight its clinical utility, though future enhancements should include longitudinal data and lifestyle factors to refine risk assessments further.

## 1 Introduction

Non-alcoholic fatty liver disease (NAFLD) is the accumulation of excessive fat in the liver in the absence of excessive alcohol consumption. It represents a manifestation of metabolic syndrome in the liver and is often associated with obesity, type II diabetes, and hyperlipidemia (1). Clinically, NAFLD may present with elevated liver enzymes, hepatomegaly, or nonspecific symptoms such as fatigue and abdominal discomfort. NAFLD ranges from simple steatosis (fat accumulation in the liver without inflammation or damage) to non-alcoholic steatohepatitis, which includes liver inflammation and damage, potentially progressing to cirrhosis or liver cancer (2). With the changes in globalization and lifestyles, NAFLD has become one of the most common chronic liver diseases, affecting approximately 25% of adults worldwide (3). The global epidemic of obesity is contributing to the rise of metabolic conditions, which in turn leads to a substantial increase in the clinical and economic burden of NAFLD (4). Several studies have demonstrated a significant correlation between the increasing prevalence and incidence of NAFLD and the mortality rates associated with liver diseases (5). According to the American Gastroenterological Association, NAFLD is projected to surpass all other causes and become the primary reason for liver transplantation in the United States by 2030 (6). However, a substantial number of individuals with NAFLD remain undiagnosed and untreated, primarily due to a lack of efficient diagnostic tools and effective pharmacological interventions. Conducting early screenings for effective interventions can significantly reduce and delay the onset of adverse prognostic events associated with NAFLD. Therefore, investigating the related risk factors and effective screening approaches for NAFLD is essential to reduce its morbidity and mortality rates.

Histopathological examination of liver biopsy has long been regarded as the gold standard for diagnosing NAFLD. However, this method has several limitations, including invasiveness, poor acceptability, and high cost (7). Furthermore, it may not accurately represent the extent of liver disease owing to the possibility of sampling error (8). Imaging methods have become increasingly accepted as noninvasive alternatives to liver biopsy in clinical practice. Ultrasonography is a widely recognized and cost-effective imaging method utilized for diagnosing hepatic steatosis, with acceptable sensitivity and specificity in detecting moderate-to-severe hepatic steatosis (9, 10). However, ultrasonography may not be suitable for monitoring NAFLD patients after therapeutic interventions given its limited capacity to accurately identify moderate steatosis, dependence on the operator’s skills, and qualitative nature without specialized picture postprocessing (11). In recent years, liver ultrasound transient elastography has emerged as an accurate and noninvasive method for assessing the degree of steatosis and fibrosis in patients with NAFLD (12). It is based on controlled attenuation parameter (CAP) and liver stiffness measures, using vibration-controlled transient elastography. A meta-analysis study found that using CAP as a tool for assessing hepatic steatosis demonstrates good diagnostic performance (AUC > 0.8) when compared with liver biopsy as the reference (13). Recently, significant efforts have been dedicated to the development of noninvasive diagnostic approaches for NAFLD. Numerous studies have developed NAFLD risk prediction models using variables such as body mass index (BMI), alanine aminotransferase (ALT), aspartate aminotransferase (AST), triglyceride (TG), total cholesterol (TC), high-density lipoprotein (HDL), and waist circumference (WC) (14, 15). Furthermore, the triglyceride glucose (TyG) index is acknowledged as an effective and simple proxy for assessing insulin resistance (IR), demonstrating considerable importance in NAFLD (16, 17). However, most of these studies used a single measurement of the TyG index to predict NAFLD risk.

With technological advancements, artificial intelligence has achieved significant breakthroughs in the medical sector. Machine learning (ML), a burgeoning aspect of artificial intelligence, is increasingly applied in healthcare data analysis to enhance clinical decision-making process (18). Despite the robust capabilities of ML approaches, which are derived from their complex models, these methods are often constrained by challenges in providing clear interpretations because of their “black-box” nature (19). Previous studies have used ML approaches to predict the risk of NAFLD (20–22). However, these studies had some limitations. Primarily, these limitations include insufficient sample sizes, which can influence the generalizability and accuracy of the models. Most researchers rely on established clinical variables to construct predictive models, overlooking the inclusion of metabolic-related indicators such as the metabolic score for IR (METS-IR) index. Creating a prediction model with a limited number of variables is crucial because employing excessive variables in ML models can deter clinicians from widely accepting the developed model.

The development of NAFLD is well established to be closely linked to IR, dyslipidemia, and obesity, particularly abdominal obesity (23, 24). Therefore, this study aims to develop and validate an interpretable ML model that predicts the probability of a patient developing NAFLD by combining the TyG index with common clinical features, elucidating the importance of features, and explaining the model through the SHapley Additive exPlanations (SHAP) method.

## 2 Materials and methods

### 2.1 Study design and participants

The Dryad database, which is funded by the National Science Foundation, serves as a repository for high-quality research data. Its primary objective is to facilitate academic exchange by protecting and promoting the reuse of research data in scientific publications. The Dryad Digital Repository website was utilized to obtain data for this investigation (https://Datadryad.org). This website provides open access to the raw data of published papers, allowing for their unrestricted reuse in secondary analysis. In accordance with the Dryad Terms of Service, we referenced the specific Dryad data package (Data from Ectopic fat obesity presents the greatest risk for incident diabetes: a population-based longitudinal study, https://datadryad.org/stash/dataset/doi:10.5061%2Fdryad.8q0p192) in this study. The raw data utilized in this study were publicly provided by Okamura et al. in 2019 (25).

To validate the prediction models generated from the Dryad database, we used data from the National Health and Nutrition Examination Survey (NHANES) that spanned the 2017–2020 cycle as part of an external validation cohort. NHANES is a comprehensive collection of surveys designed to assess the health and nutritional status of the noninstitutionalized general population throughout the United States. The NHANES study protocol was granted permission by the National Center for Health Statistics Research Ethics Review Board, and all participants were thoroughly informed and provided their consent.

### 2.2 Data collection

The Dryad data contained the results of physical examinations conducted at Murakami Memorial Hospital between 2004 and 2015. The baseline data excluded participants with alcoholic fatty liver disease and viral hepatitis. NAFLD was diagnosed on the basis of the findings of abdominal ultrasonography performed by trained technicians.

The clinical information extracted included sex, age, BMI, WC, history of alcohol consumption, visceral fat obesity (WC≥90 in men, ≥80 in women), obesity (BMI≥25), TG, TC, HDL, AST, ALT, gamma-glutamyl transpeptidase (GGT), systolic blood pressure, diastolic blood pressure, HbA1c, and fasting plasma glucose (FPG). Among them, 551 cases were excluded because of heavy alcohol consumption and missing values.

**Figure 1:**
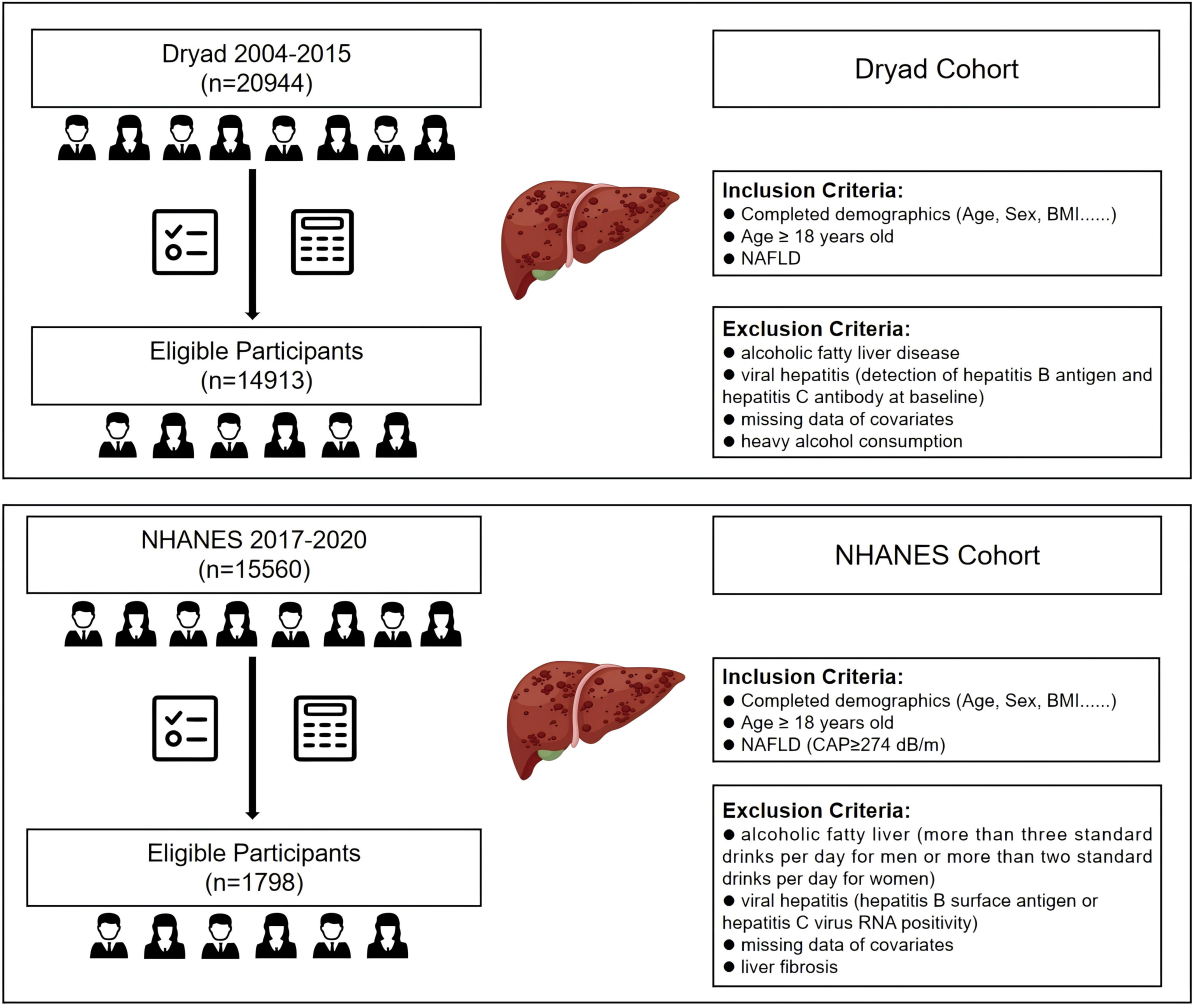
Flow diagram of the inclusion and exclusion criteria for the collection of data on NAFLD patients in the Dryad and NHANES cohorts. NAFLD, non-alcoholic fatty liver disease.

The data for external validation were extracted from the 2017–2020 cycle released by NHANES. This study consisted of individuals aged at least 18 years who have undergone liver ultrasound transient elastography to obtain measurements for CAP and liver stiffness measurement (LSM). According to the literature, CAP≥274 dB/m was considered an indicator of liver steatosis, and LSM≥8 kPa indicated the presence of liver fibrosis (26, 27). To ensure the integrity and validity of our findings, we implemented rigorous exclusion criteria. This study excluded participants with a history of excessive alcohol consumption, defined as more than 21 standard drinks per week for males or more than 14 standard drinks per week for females. Individuals with positive serological markers for the hepatitis B or C virus, diagnosed cases of hepatitis B or C by a physician, and those with liver fibrosis were also excluded. Moreover, the analysis excluded individuals with missing data for essential covariates, such as BMI, WC, AST, ALT, GGT, HDL, and FPG. Ultimately, we obtained 14,913 and 1,798 participants from the Dryad and NHANES databases, respectively (**Figure 1**).

### 2.3 Definitions of TyG, TyG–WC, TyG-BMI, TG/HDL, and METS-IR score

TyG, TyG–WC, TyG-BMI, TG/HDL, and METS-IR were calculated using the following equations (28–32):


TyG = ln [(TG (mg/dL) × FPG (mg/dL))/2]

TyG−WC = TyG × WC

TyG−BMI = TyG × BMI

TG/HDL = TG (mg/dL) / HDL (mg/dL)

METS−IR = ln [(2 × FPG (mg/dL))+ TG (mg/dL)] × BMI / ln (HDL (mg/dL))

### 2.4 Model development and comparison

**Figure 2:**
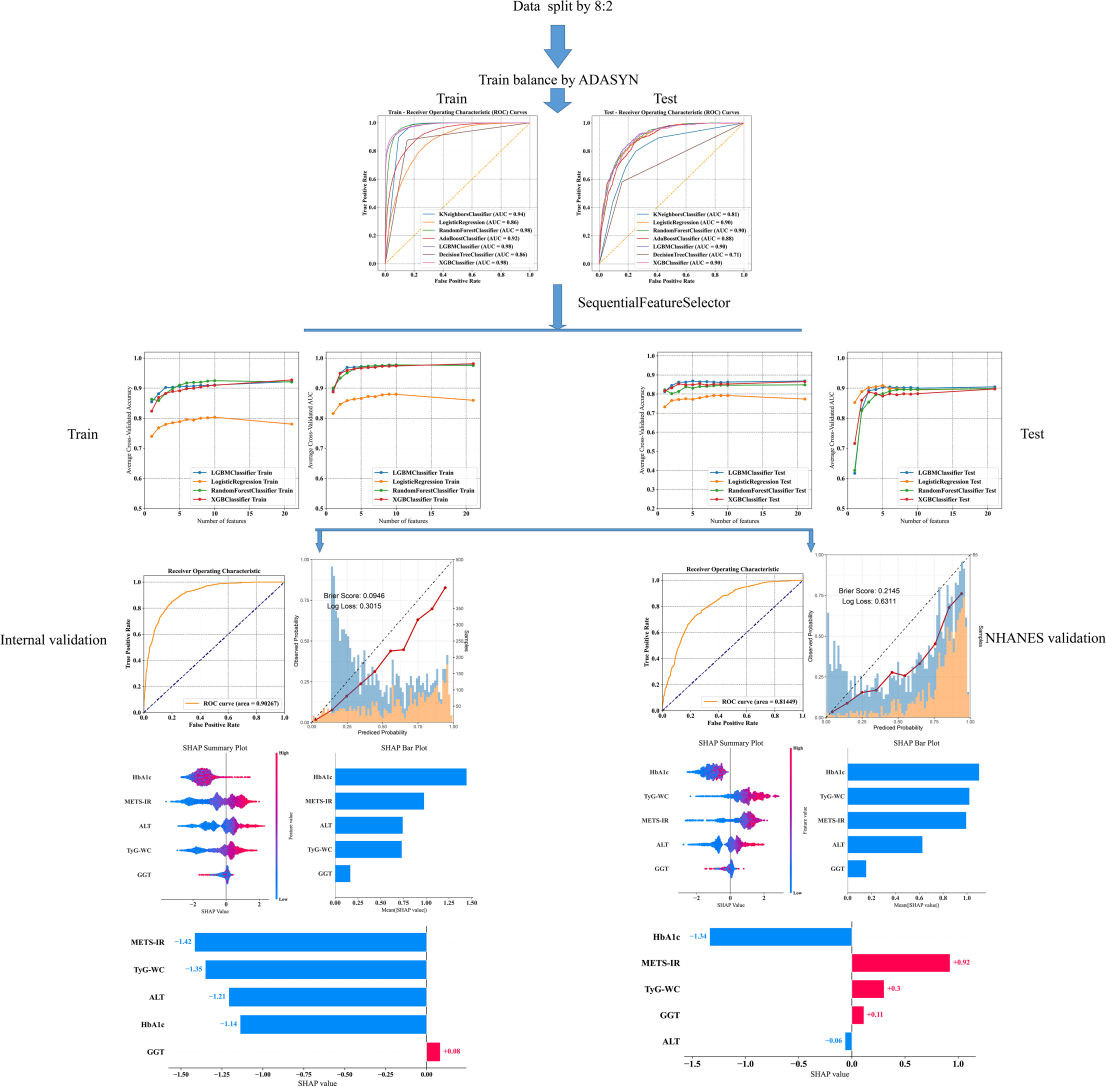
Machine learning flowchart of this study.

The overall ML workflow chart is illustrated in **Figure 2**. Data from the Japanese cohort were divided, with 80% allocated for training and 20% for internal validation, to prevent the issue of overfitting. Features from the Japanese dataset were cross-referenced with those from NHANES, ultimately identifying 21 common features for the development of a predictive model. Seven ML models, namely, *k*-nearest neighbor (KNN), logistic regression (LR), random forest (RF), adaptive boosting (AdaBoost), light gradient boosting machine (LGBM), decision tree (DT), and extreme gradient boosting (XGBoost), were employed to predict NAFLD. Ten-fold cross-validation was performed in the training queue to validate the prediction model and avoid overfitting. Several commonly used evaluation metrics, including the area under the receiver-operating-characteristic (ROC) curve (AUC), sensitivity, specificity, accuracy, and F1 score, were utilized to assess the reliability of these models.

### 2.5 Feature selection and model explanation

Features for the selected ML models were incrementally added using the “SequentialFeatureSelector” method until no significant increase in AUC was observed. Grid search combined with manual fine-tuning was employed to determine the final hyperparameters to optimize the predictive model. To ensure the robustness and independence of the features in our model, we calculated the variance inflation factor (VIF) for the selected variables in the final model. This assessment helped us identify potential multicollinearity issues, ensuring that each feature independently contributes to the predictive accuracy. A VIF value greater than 10 can be used as a strong indicator of multicollinearity (33). Interpreting ML models is challenging. SHAP provides global and local explanations for model interpretation. Global explanations offer consistent and precise attribution values for each feature in the model, illustrating the association between input features and the outcome. Local explanations demonstrate specific predictions for individual NAFLD patients by inputting specific data.

### 2.6 Statistical analysis

Data analyses were conducted using Python version 3.9.0, accessible at https://www.python.org, with the following packages and their versions: scikit-learn (v1.2.2), shap (v0.45.1), xgboost (v1.7.1), imblearn (v0.8.1), and lightgbm (v3.3.3). Given the non-normal distribution of the data, continuous variables were expressed as the median and interquartile range. A comparison between the two groups was conducted using the Wilcoxon rank-sum test. Categorical variables were presented as frequencies and percentages, and between-group comparisons were performed using the chi-square test. Decision curve analysis (DCA) and precision–recall curve analysis were performed using R version 4.1.3 (https://www.r-project.org). A two-tailed P value of less than 0.05 was considered statistically significant.

## 3 Results

### 3.1 Baseline characteristics of the study population

A total of 14,913 study participants, aged 18–79 years, were included in this study. Of these participants, 7,879 (52.8%) were males, and 7,034 (47.2%) were females. The remaining characteristics are presented in **Table 1**. Statistically significant differences in all variables were observed between the NAFLD and non-NAFLD groups (P < 0.05). A comparison of demographic and clinical variables among the NHANES cohort is provided in **Supplementary Table 1**.

**Table 1:** Demographic and clinical characteristics of study population in the Dryad cohort.

Characteristic	Non-NAFLD (N = 12,293)	NAFLD (N = 2,620)	p-value
Sex			<0.001
Female	6,548 (53%)	486 (19%)	
Male	5,745 (47%)	2,134 (81%)	
Age	42 (36, 50)	44 (38, 51)	<0.001
BMI(kg/m^2^)	21.2 (19.5, 23.0)	25.1 (23.4, 27.2)	<0.001
WC(cm)	74 (68, 80)	86 (81, 91)	<0.001
Visceral fat obesity			<0.001
No	11,296 (91.9%)	1674 (64%)	
Yes	997 (8.1%)	946 (36%)	
Obesity			<0.001
No	11,244 (91.5%)	1,275 (49%)	
Yes	1,049 (8.5%)	1,345 (51%)	
ALT(U/L)	15 (12, 20)	27 (20, 39)	<0.001
AST(U/L)	17.0 (14.0, 20.0)	21.0 (17.0, 26.0)	<0.001
GGT(U/L)	14 (11, 19)	23 (17, 34)	<0.001
HDL(mmol/L)	1.48 (1.24, 1.76)	1.14 (0.99, 1.34)	<0.001
TC(mmol/L)	4.99 (4.45, 5.59)	5.43 (4.86, 6.00)	<0.001
TG(mmol/L)	0.65 (0.45, 0.96)	1.25 (0.87, 1.80)	<0.001
TyG	7.88 (7.50, 8.29)	8.59 (8.22, 8.95)	<0.001
TyG–BMI	167 (149, 187)	216 (197, 238)	<0.001
TyG–WC	582 (519, 650)	737 (678, 793)	<0.001
TG/HDL	1.00 (0.62, 1.65)	2.47 (1.57, 3.89)	<0.001
METS-IR	29 (26, 33)	38 (34, 43)	<0.001
HbA1c(%)	5.10 (4.90, 5.40)	5.30 (5.10, 5.50)	<0.001
FPG(mmol/L)	5.11 (4.83, 5.38)	5.38 (5.16, 5.66)	<0.001
SBP(mmHg)	111 (102, 121)	123 (113, 133)	<0.001
DBP(mmHg)	70 (63, 76)	77 (71, 84)	<0.001

BMI, body mass index; WC, waist circumference; ALT, alanine aminotransferase; AST, aspartate aminotransferase; GGT, gamma-glutamyl transpeptidase; HDL, high-density lipoprotein; TC, total cholesterol; TG, triglyceride; FPG, fasting plasma glucose; SBP, systolic blood pressure; DBP, diastolic blood pressure.

### 3.2 Model development and performance comparison

**Figure 3:**
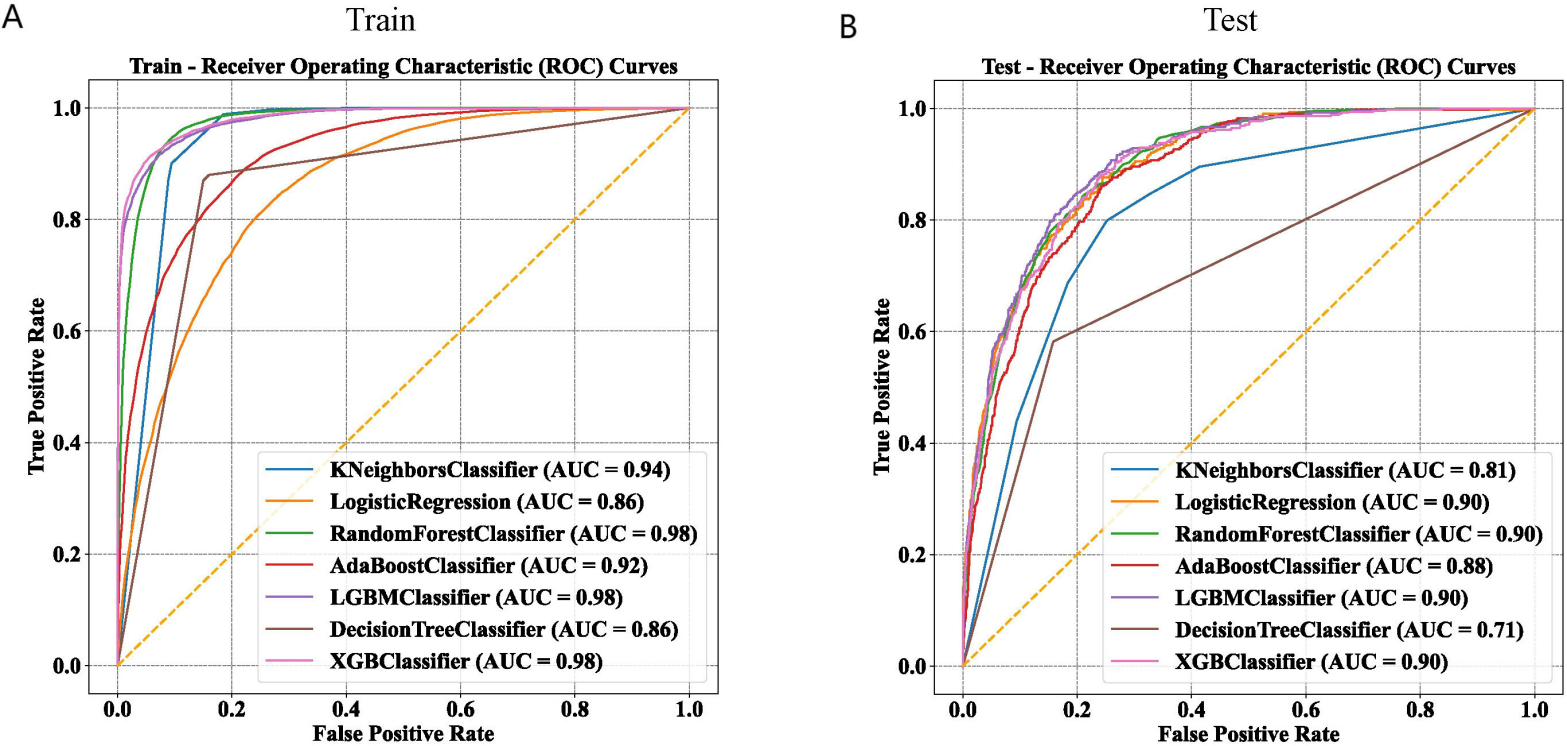
Comparison of machine learning models on training and test datasets using ROC curves. **(A)** ROC curves of seven machine learning models in the training set. **(B)** ROC curves of seven machine learning models in the test set.

In this study, 21 clinical variables were utilized to develop 7 ML models for predicting the risk of NAFLD. Among the seven models, the RF, LGBM, and XGBoost models achieved an AUC greater than 0.95 in the training set (**Figure 3A**). In the internal test set, the LR, LGBM, RF, and XGBoost models all recorded an AUC of 0.90 (**Figure 3B**).

The discriminative performance of these seven models is presented in **Tables 2** and **3**. The results indicated that the LGBM and XGBoost models achieved the highest overall performance, with high accuracy, precision, recall, F1 scores, and AUC values, in the training and test sets. The RF model also performed well, especially in terms of recall and AUC. The KNN model showed high recall but low precision and F1 scores in the test set. The LR, AdaBoost, and DT models showed moderate performance across different metrics. The Matthews correlation coefficient and kappa values supported these observations, highlighting the robustness of the LGBM and XGBoost models in predicting NAFLD. These findings suggest that ensemble methods, particularly LGBM, are highly effective for predicting NAFLD, providing valuable insights for clinical decision-making and patient management.

**Table 2:** Performance of the machine learning models for NAFLD prediction in the training set.

Model	Accuracy	Precision	Recall	F1	Kappa	MCC	AUC
KNN	0.867	0.796	0.997	0.885	0.733	0.759	0.943
LR	0.780	0.780	0.794	0.787	0.559	0.559	0.858
RF	0.921	0.887	0.968	0.926	0.841	0.844	0.975
AdaBoost	0.836	0.813	0.883	0.847	0.671	0.674	0.919
LightGBM	0.922	0.923	0.924	0.924	0.844	0.844	0.980
DT	0.864	0.859	0.879	0.869	0.728	0.728	0.864
XGboost	0.925	0.928	0.926	0.927	0.851	0.851	0.982

NAFLD, non-alcoholic fatty liver disease; AUC, area under the receiver-operating-characteristic curve; KNN, k-nearest neighbor; LR, logistic regression; RF, random forest; AdaBoost, adaptive boosting; LightGBM, light gradient boosting machine; DT, decision tree; XGboost, eXtreme gradient boosting; MCC, Matthews correlation coefficient.

**Table 3:** Performance of the machine learning models for NAFLD prediction in the testing set.

Model	Accuracy	Precision	Recall	F1	Kappa	MCC	AUC
KNN	0.757	0.399	0.799	0.533	0.392	0.435	0.815
LR	0.776	0.428	0.870	0.574	0.445	0.496	0.899
RF	0.848	0.546	0.731	0.625	0.533	0.541	0.898
AdaBoost	0.782	0.433	0.824	0.568	0.440	0.481	0.883
LightGBM	0.868	0.613	0.650	0.631	0.551	0.551	0.904
DT	0.797	0.436	0.582	0.498	0.374	0.380	0.712
XGboost	0.866	0.619	0.590	0.604	0.523	0.524	0.896

NAFLD, non-alcoholic fatty liver disease; AUC, area under the receiver-operating-characteristic curve; KNN, k-nearest neighbor; LR, logistic regression; RF, random forest; AdaBoost, adaptive boosting; LightGBM, light gradient boosting machine; DT, decision tree; XGboost, eXtreme gradient boosting; MCC, Matthews correlation coefficient.

### 3.3 Identification of the final model

**Figure 4:**
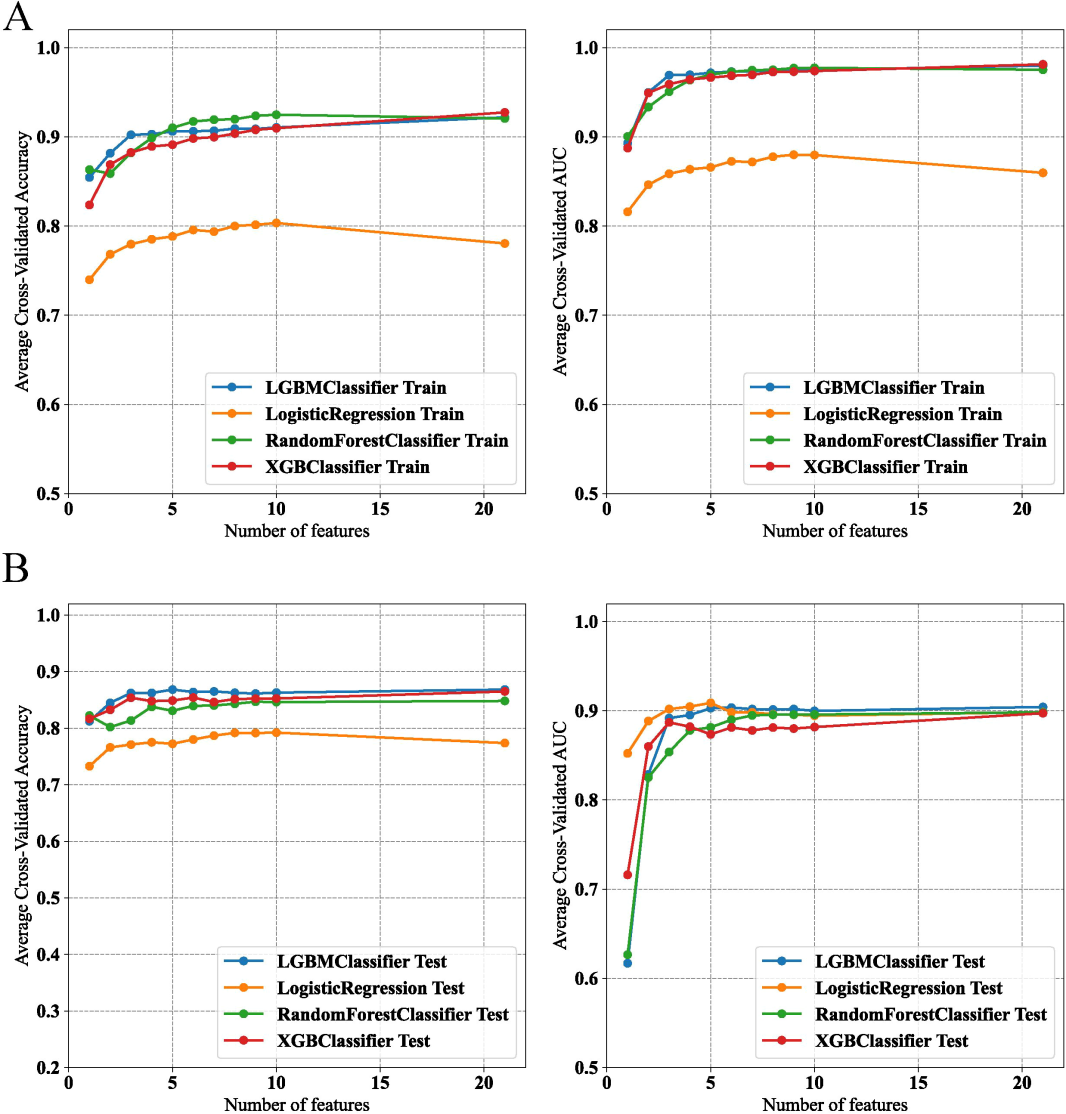
Performance evaluation of machine learning models on feature selection in training and test datasets. **(A)** Model accuracy and AUC for various classifiers in the training set. **(B)** Model accuracy and AUC for various classifiers in the test set.

The final model was determined during the feature addition process among the top four models. As shown in **Figures 4A, B**, the changes in accuracy and AUC for the training set and internal test set across the four models indicated that the LGBM model almost consistently maintained the best predictive ability among them. Additionally, when the number of features was around five, no significant increases in accuracy and AUC occurred, with the LGBM model performing the best.

On the basis of the results of feature selection, the LGBM model incorporating five features—ALT, GGT, TyG–WC, METS-IR, and HbA1c—was selected as the final model for further analysis. Further validation of the model’s robustness was conducted by calculating the VIF for these features. All these variables had VIF values less than 10, specifically: ALT (VIF=1.42), GGT (VIF=1.38), TyG–WC (VIF=5.16), METS-IR (VIF=4.93), and HbA1c (VIF=1.03). That is, no significant multicollinearity existed among the variables in the final model, ensuring the independence of each predictor in our analysis.

**Figure 5:**
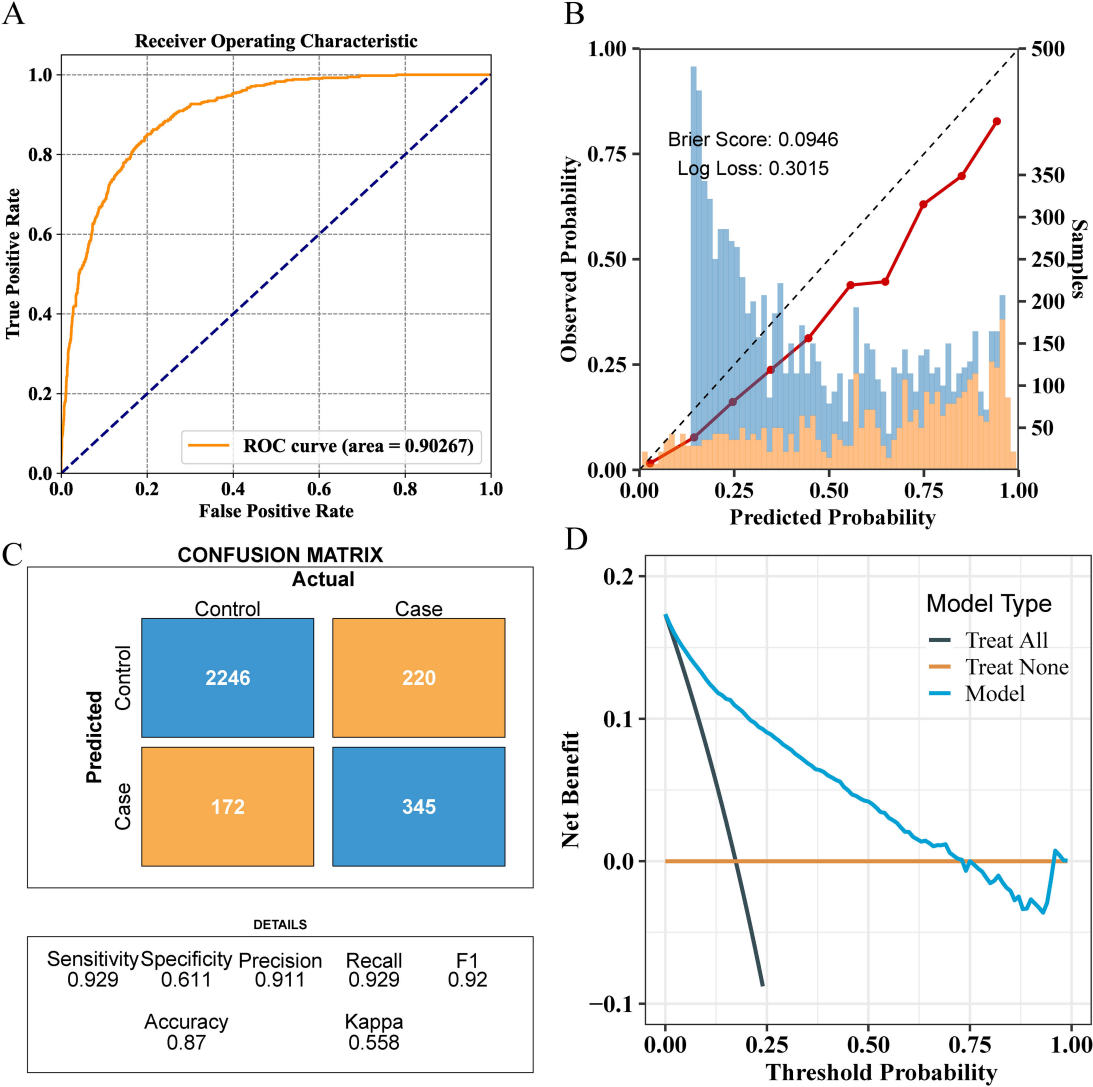
Comprehensive evaluation of the final model’s performance on the training set. **(A)** ROC curve illustrating the model’s diagnostic ability. **(B)** Calibration plot with the Brier score and Log loss. Bars indicate the group with NAFLD (orange) and the control group (blue) per interval of predicted probability. **(C)** Confusion matrix detailing actual vs. predicted classifications. **(D)** Decision curve analysis showing the net benefit across different threshold probabilities.

To optimize the prediction model, we employed “RandomizedSearchCV” for random grid search, combined with manual fine-tuning to determine the final hyperparameters (“subsample”: 1.0, “n_estimators”: 100, “max_depth”: −1, “learning_rate”: 0.1, “colsample_bytree”: 1.0). The final LGBM model achieved an AUC of 0.90 in the internal validation set for predicting whether patients have NAFLD (**Figure 5A**). The calibration curve yielded a Brier score of 0.0946 and a Log loss of 0.3015 (**Figure 5B**). The confusion matrix revealed the model’s performance in actual classification, with an accuracy of 0.87, a sensitivity of 0.929, and a specificity of 0.611 (**Figure 5C**). In the internal test cohort, DCA showed that when the threshold probability exceeded 25%, the average net benefit of using the LGBM model to predict NAFLD was superior to that of using strategies for treating all or treating none (**Figure 5D**).

### 3.4 External validation of the final model

**Figure 6:**
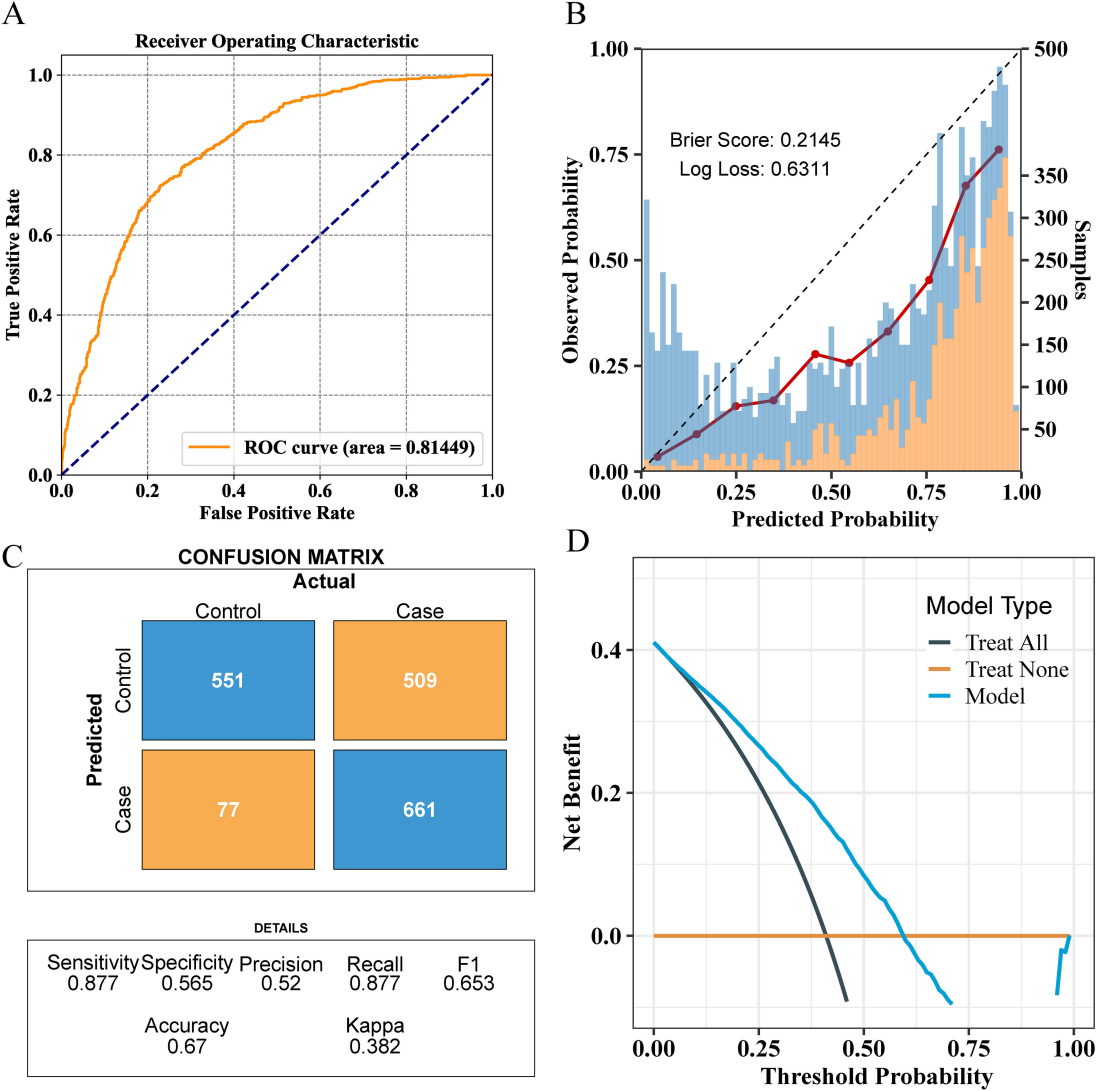
Comprehensive evaluation of the final model’s performance on the validation set. **(A)** ROC curve illustrating the model’s diagnostic ability. **(B)** Calibration plot with the Brier score and Log loss. Bars indicate the group with NAFLD (orange) and the control group (blue) per interval of predicted probability. **(C)** Confusion matrix detailing actual vs. predicted classifications. **(D)** Decision curve analysis showing the net benefit across different threshold probabilities.

For external validation, the final model achieved an AUC of 0.81, indicating good performance in external validation (**Figure 6A**). The calibration curve had a Brier score of 0.2145 and a Log loss of 0.6311 (**Figure 6B**). The confusion matrix indicated an accuracy of 0.67, a sensitivity of 0.877, and a specificity of 0.565 (**Figure 6C**). In the NHANES external test cohort, DCA showed that when the threshold probability exceeded 45%, the average net benefit of using the LGBM model to predict whether patients have NAFLD was superior to that of using strategies for treating all or treating none (**Figure 6D**).

### 3.5 Model explanation

**Figure 7:**
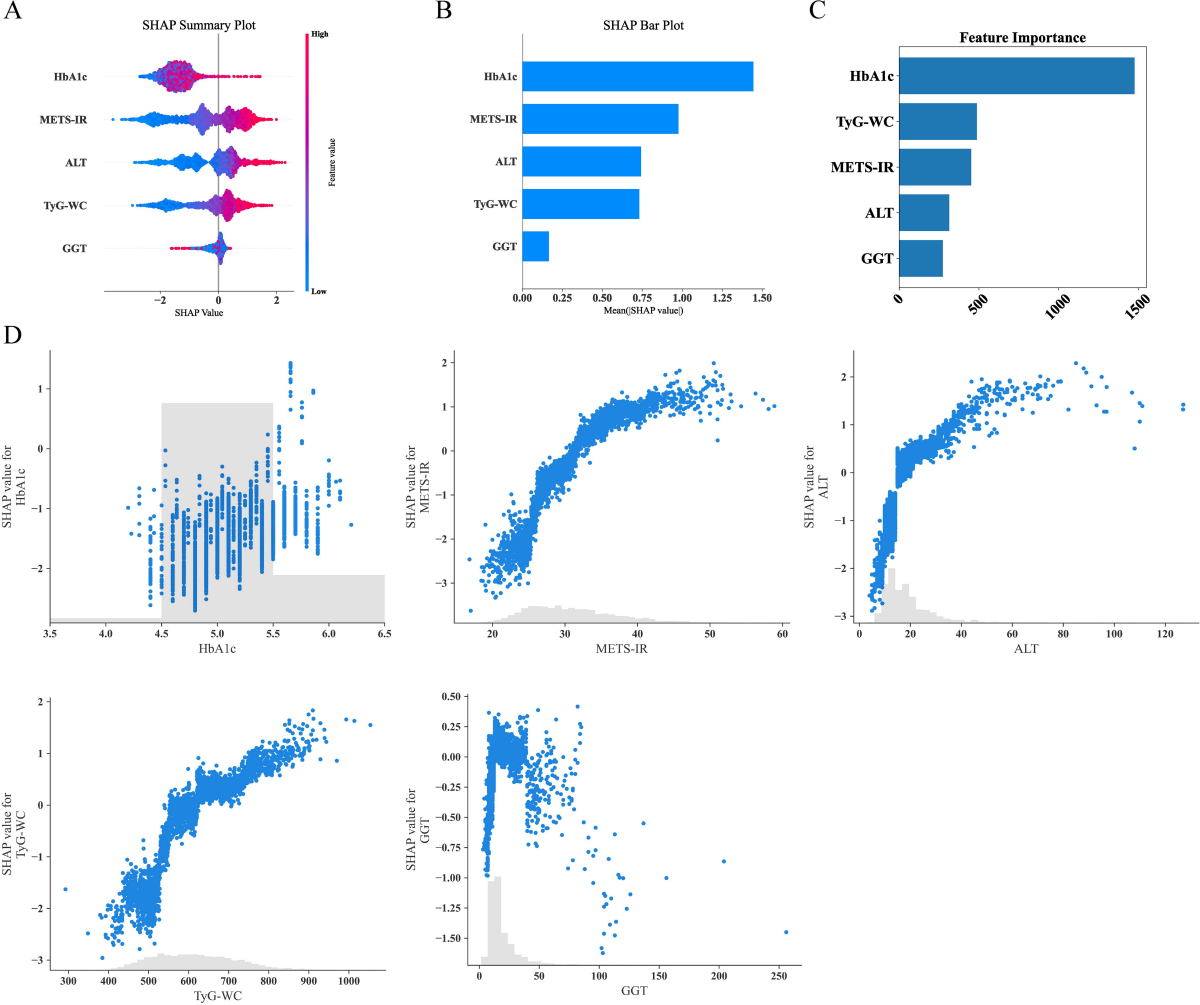
Analysis of feature importance and relationships in predictive modeling. **(A)** SHAP summary plot showing the effects of features on model output. **(B)** SHAP bar plot illustrating the mean SHAP values for each feature. **(C)** Feature importance ranking based on total SHAP values. **(D)** Detailed SHAP value plots for individual features, demonstrating their contribution to model predictions. SHAP, SHapley Additive explanations.

**Figure 8:**
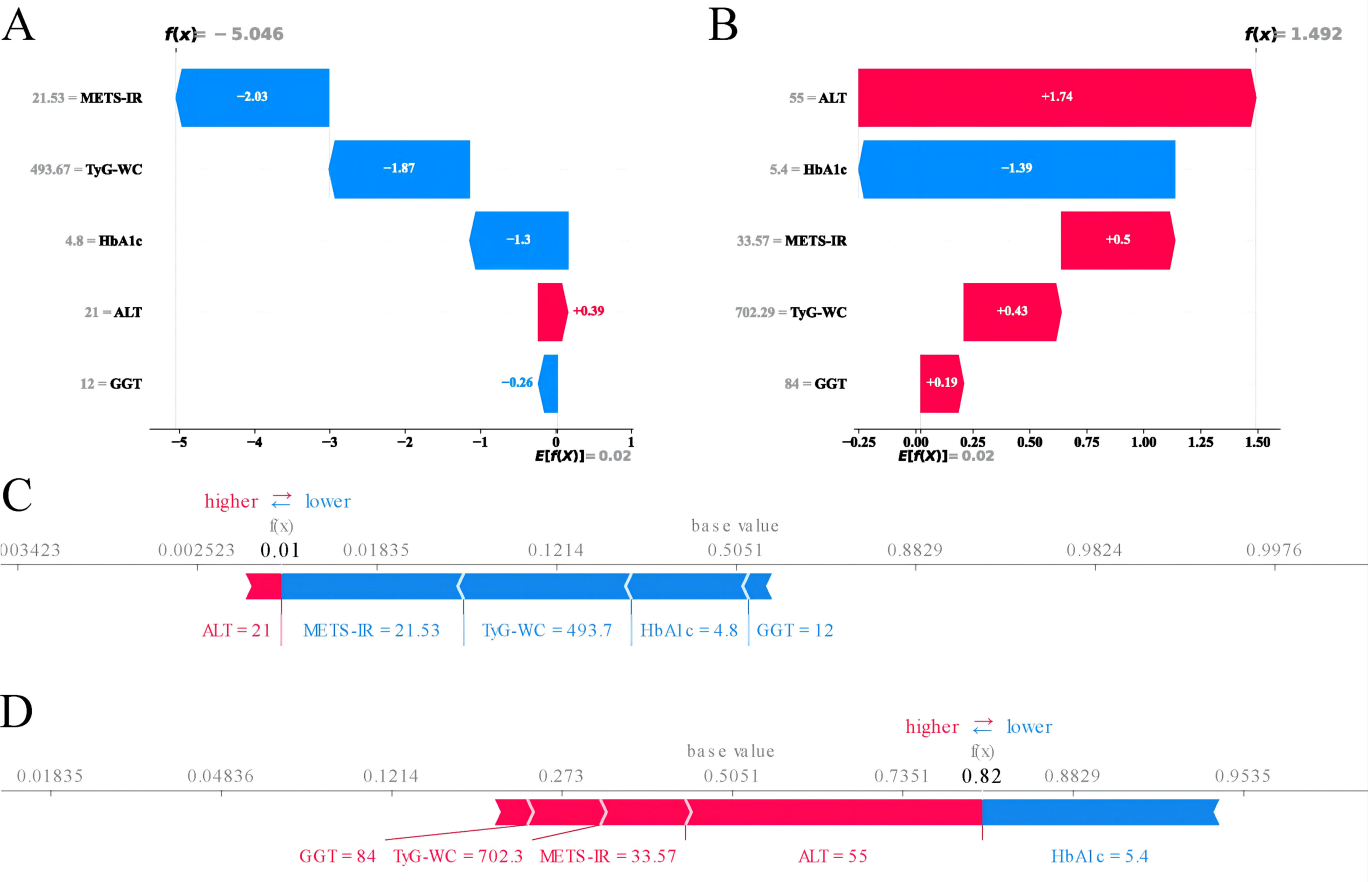
Machine learning model analysis using biochemical markers to predict NAFLD. **(A)** SHAP values for features suggesting a non-NAFLD prediction. **(B)** SHAP values for features suggesting an NAFLD prediction. **(C)** Waterfall plot illustrating the cumulative effect of features on the model’s output starting from the base value for a non-NAFLD prediction. **(D)** Waterfall plot showing the cumulative effect of features for an NAFLD prediction. SHAP, SHapley Additive explanations; NAFLD, non-alcoholic fatty liver disease.

Given that clinicians hardly accept a prediction model that is not directly explainable and interpretable, the SHAP method is utilized to interpret the output of the final model by calculating the contribution of each variable to the prediction. The SHAP summary plot, SHAP bar plot, feature importance graph (**Figures 7A–C**), and dependence plot (**Figure 7D**) delineated the contributions of the five predictors within the LGBM model. The SHAP summary plot revealed the specific contributions of these features to model predictions, with HbA1c showing the highest mean absolute SHAP value, indicating its strong influence on the model. SHAP values above zero indicate a high risk of developing NAFLD, whereas values below zero indicate a low risk. For example, high METS-IR (red) typically results in SHAP values greater than zero, implying a high risk of NAFLD in patients with high METS-IR scores. **Figure 7B** portrays the feature rankings based on the average absolute SHAP value. HbA1c, METS-IR, ALT, and TyG–WC emerged as the four most influential variables in predictive power. Elevated levels of HbA1c, METS-IR, ALT, and TyG–WC indicate an increased likelihood of NAFLD. The dependence plot revealed a significant effect of HbA1c in the range of 5.0–5.5 on model predictions. The local explanation analyzed how a specific prediction was made for an individual by incorporating their individualized input data. **Figure 8** displays the results of an ML model that used individualized biochemical marker data to predict NAFLD. According to the prediction model, the result shown in **Figure 8A**, f(x) = −5.046, likely indicated a high probability of being non-NAFLD. Conversely, the result in **Figure 8B**, f(x) = 1.492, indicated a relatively high likelihood of NAFLD. **Figures 8C, D** further detail the contribution of each feature to the model’s predictive output, showing how increases or decreases in feature values specifically affect the prediction results through different baseline values. The SHAP summary plot (**Supplementary Figure 1**) depicts the role of five features in the NHANES external validation set in predicting NAFLD.

## 4 Discussion

The increasing prevalence of NAFLD worldwide has prompted the need for reliable risk prediction models that can aid in the early identification and prevention of the disease as the most effective approach to improving its outcomes. In this study, a large-scale physical examination population of 14,913 subjects was used to establish 7 ML predictive models based on 21 clinical variables. Among these models, the internal and external validation sets demonstrated that the LGBM model exhibited exceptionally high predictive accuracy, with an AUC of 0.90 in the internal validation set and 0.81 in the external validation set.

In this study, LGBM outperformed all other compared ML models. LGBM, a tree-based gradient boosting framework, is designed for efficiency and scalability, making it particularly suitable for handling large-scale and complex datasets (34). One of LGBM’s advantages is its use of gradient-based one-side sampling (GOSS) and exclusive feature bundling (EFB), which optimize the calculation of information gain and reduce model complexity in high-dimensional data (35).GOSS effectively retains high-gradient samples while reducing the sampling rate of low-gradient ones, proving especially effective in addressing the imbalance issues commonly found in clinical datasets (36). EFB, by combining mutually exclusive features, reduces the dimensionality of the model, thereby improving computational efficiency without significant loss of information (36). Moreover, LGBM’s high memory use efficiency allows it to maintain high performance even with limited hardware resources (37). These features, along with LGBM’s capability to handle sparse data (common in clinical datasets owing to missing values) and built-in support for categorical features, likely explain its superior performance compared with models such as LR, DT, and RF, which may not handle dimensionality and model complexity as effectively (38). Therefore, the choice of the LGBM model was not only based on its high performance but also due to its advantages in interpretability and operability, providing a powerful tool for clinical decision-making.

In our study, we identified five key predictive variables: ALT, GGT, TyG–WC, METS-IR, and HbA1c, all of which are closely associated with metabolic abnormalities. The liver, a vital organ for metabolism, regulates the metabolism of lipids and glucose. The presence of these predictive variables suggests a strong correlation with metabolic syndrome, a cluster of conditions, including increased blood pressure, high blood sugar, excess body fat around the waist, and abnormal cholesterol or TG levels, which can increase the risk of heart disease, stroke, and diabetes (39, 40). Elevated levels of ALT and GGT are indicative of liver stress or damage, possibly due to lipid accumulation, a common occurrence in metabolic syndrome (41). The TyG index is a valuable biomarker used to assess IR, a pivotal component in the pathophysiology of various metabolic disorders (28). Building on the foundation of the TyG index, TyG–WC incorporates WC with the TyG index to enhance the predictive power for metabolic abnormalities, particularly those related to obesity and central fat distribution. Research has shown that elevated TyG–WC values are associated with an increased prevalence of NAFLD (42). METS-IR is another key metric for assessing metabolic syndrome and IR. Elevated METS-IR levels are often found in individuals with NAFLD, indicating a strong link between IR and liver fat accumulation (43). HbA1c, as a measure of long-term glucose control, is particularly relevant in the context of metabolic syndrome and NAFLD because of its reflection of chronic hyperglycemia, which can exacerbate IR and contribute to liver fat accumulation (44, 45). The research findings underscore the critical role of these key variables in predicting NAFLD, particularly given their strong links to metabolic abnormalities.

Given the limitations of imaging in the diagnosis of NAFLD, several studies have explored the possibility of using biomarkers of steatosis for prediction, including the fatty liver index (FLI), the hepatic steatosis index (HSI), and the NAFLD-liver fat score (NAFLD-LFS) (46–48). In the study by Atabaki-Pasdar et al., a new model for predicting NAFLD was developed using various ML methods to integrate genetic, transcriptomic, proteomic, metabolomic, and clinical variables, achieving an AUC of 0.84 with the inclusion of all omics and clinical variables (49). The study also compared the predictive capabilities of FLI, HSI, and NAFLD-LFS, revealing that the predictive power of a multiomic variable model significantly surpasses that of a single steatosis biomarker. In the study by Kouvari et al., the diagnostic performance of existing and new noninvasive liver disease indices was validated via liver biopsy (50). The research results showed that the index of non-alcoholic steatohepatitis (ION) performed best in distinguishing patients with NAFLD from the control group, with an AUC of 0.894. The FLI, NAFLD-LFS, and ION indices provide important references for the diagnosis of NAFLD. Compared to these indices, our model demonstrates superior performance in two key metrics: sensitivity and AUC. Specifically, our model achieves a sensitivity of 0.929 and an AUC of 0.902, significantly surpassing FLI (sensitivity: 0.707, AUC: 0.701), NAFLD-LFS (sensitivity: 0.709, AUC: 0.871), and ION (sensitivity: 0.710, AUC: 0.894). However, it is noteworthy that despite its excellent performance in sensitivity and overall diagnostic capabilities, our model still exhibits lower specificity compared to NAFLD-LFS and ION (**Supplementary Table 2**). This indicates that there is still room for improvement in reducing false positives in our model.

In 2023, multiple international liver disease associations released the “Multi-Society Delphi Consensus on the New Nomenclature for Fatty Liver Disease,” ultimately proposing the renaming of NAFLD to metabolic dysfunction-associated steatotic liver disease (MASLD) (51). This evolution of terminology reflects a deeper understanding of NAFLD, acknowledging its close association with metabolic syndrome, including diabetes, obesity, and dyslipidemia. A recent study has shown that nearly 99% of NAFLD patients also meet the diagnostic criteria for MASLD, indicating that the natural histories of the two are nearly identical (52). Additionally, the differences in prevalence and disease progression between NAFLD and MASLD are minimal. In our study, we used the term NAFLD, primarily based on the timeframe of our data collection and analysis. Future research should broadly adopt the new term MASLD and develop and validate new noninvasive diagnostic tools that systematically consider the multifaceted aspects of metabolic dysfunction. This line of research will aid in accurately differentiating the various stages of the disease and tailoring diagnostic and treatment strategies to individual metabolic profiles, fully reflecting the metabolic health of patients.

In recent years, numerous studies have developed predictive models for NAFLD, achieving good predictive performance but also exhibiting notable limitations. Motamed et al. developed an LR model for predicting NAFLD, incorporating FLI, with an AUC of 0.866 (53). However, the study did not utilize advanced ML methods, which are excellent at handling complex data interactions and nonlinear relationships, potentially limiting the model’s predictive power. Peng et al. developed and validated five ML models for predicting NAFLD using variables such as visceral adiposity index, abdominal circumference, BMI, and ALT (20). The study showed that the XGBoost model presented the best predictive performance, with an AUC of 0.938. Although the study used magnetic resonance imaging-proton density fat fraction for external validation, which is the gold standard method, one of the main limitations of the study was its relatively small sample size, which could affect the extrapolation ability of the model. Cao et al. conducted a longitudinal cohort study using 22,140 participants from the Beijing Health Management Cohort to develop ML models for predicting NAFLD (14). Key predictive variables included AST, cardiometabolic index, BMI, ALT, and TyG index. However, the study relied on a single cohort, which may affect the generalizability of the findings. Compared with the models in these studies, our final model performed well in internal and external validations. Although our model demonstrated strong performance with datasets from Japan and the United States, noticeable differences in predictive capability between the two datasets remain, potentially due to genetic and population-specific factors. As highlighted in the study by Takahashi et al., different genetic backgrounds may significantly influence the performance of NAFLD diagnostic models (54). In addition to our research findings, Noureddin et al. used NHANES data from 2017 to 2018 and applied ML techniques to predict NAFLD identified through liver ultrasound transient elastography, demonstrating the efficacy of this method in improving the accuracy of disease predictions (55).

Our research has several significant advantages. First, our study utilized data from a large-scale physical examination population of 14,913 subjects, which is much larger than those of most previous studies. This large sample size not only increased the statistical power of the model but also improved the reliability and generalizability of the results. Second, our model included biochemical and metabolic indicators, with the introduction of new indicators such as TyG–WC and METS-IR, enhancing the model’s ability to predict metabolic syndrome and IR. Third, ML algorithms demonstrated excellent performance in handling complex data structures and large datasets. Among them, the LGBM model exhibited exceptionally high predictive accuracy, indicating its significant advantage in managing complex data and multivariable relationships compared with other ML methods. Fourth, we validated the model’s performance not only on internal datasets but also on external datasets, demonstrating its robust generalizability. This aspect was often overlooked in many previous studies, leading to suboptimal performance in practical applications. Lastly, we used SHAP to visualize the effect of each predictive variable on the model’s output. This visualization helped us understand the contribution of each variable to the prediction of NAFLD, providing valuable insights into the model’s decision-making process. Overall, our paper presents a highly reliable and accurate predictive model for NAFLD, with significant improvements over previous models in terms of sample size, variable integration, the application of advanced ML techniques, and comprehensive validation.

This study has several limitations. First, regarding the diagnosis of NAFLD, ultrasound was used for internal validation, while liver elastography was used externally. Ultrasound, widely used for preliminary clinical diagnosis, has low accuracy in quantifying fat, especially in obese patients (56). By contrast, although liver ultrasound transient elastography is more sensitive and accurate in diagnosing hepatic steatosis, it is not considered the gold standard for diagnosing NAFLD (12). Differences in diagnostic tools may lead to disparities in model predictive performance, affecting the model’s applicability and accuracy in different clinical settings. Second, although the study considered various clinical variables, it did not include important lifestyle factors that affect NAFLD risk, such as dietary habits and physical activity levels (57). These factors have a direct and significant effect on the development of NAFLD, and their absence may limit the model’s comprehensiveness and precision in predicting individual risks. Future research should consider including these variables to enhance the model’s predictive accuracy and clinical utility. Third, the study used baseline data to predict the risk of NAFLD without considering physiological and behavioral changes over time. The development of NAFLD is influenced by various factors, including age, weight gain, medication use, and other changes in health conditions. Although baseline data provide a basis for assessing initial individual risk, these preliminary assessments may no longer be accurate as time progresses. Therefore, future studies should consider using longitudinal data to track changes in individual health conditions, allowing for the construction of a more dynamic and real-time risk predictive model.

## 5 Conclusion

This study conducts a comprehensive analysis of NAFLD risk prediction using advanced ML algorithms, emphasizing the exceptional predictive performance of the LGBM model during testing. Utilizing a large-scale physical examination population in Japan and the NHANES database for external validation, this research achieves improvements in statistical power, reliability, and generalizability compared with previous studies. It identifies key predictive variables, such as ALT, GGT, TyG–WC, METS-IR, and HbA1c, highlighting their strong association with metabolic abnormalities and their crucial role in the prediction model. The use of SHAP values to interpret the contributions of these variables enhances the depth of understanding and increases the transparency and applicability of the model in clinical settings. Therefore, our research results can provide a reliable reference for the early identification of NAFLD in clinical practice.

## Data Availability

Publicly available datasets were analyzed in this study. This data can be found here: This study analyzed datasets that are publicly available. The Dryad data can be accessed at the Dryad Data Repository: http://datadryad.org/; the NHANES data are available at the following URL: https://wwwn.cdc.gov/nchs/nhanes/Default.aspx. The code used in the article and its detailed documentation can be found at the following link: https://gitee.com/g39300454/ml-code.
