# Supplementary material for: Advancing non-alcoholic fatty liver disease prediction: a comprehensive machine learning approach integrating SHAP interpretability and multi-cohort validation
Source: Front Endocrinol (Lausanne). 2024 Oct 8;15:1450317. doi: 10.3389/fendo.2024.1450317 (PMC11493712; doi:10.3389/fendo.2024.1450317)
Supplement: Supplementary file 1 [file DataSheet1.docx]

Supplementary Material

# Supplementary Figures and Tables

**Supplementary Figure 1.** Using the NHANES database to validate the importance and relationships of features in predictive modeling. **(A)** SHAP summary plot showing the impact of features on model output. **(B)** SHAP bar plot illustrating the mean SHAP values for each feature. **(C)** Detailed SHAP value plots for individual features, demonstrating their contribution to model predictions. SHAP, SHapley Additive explanations.


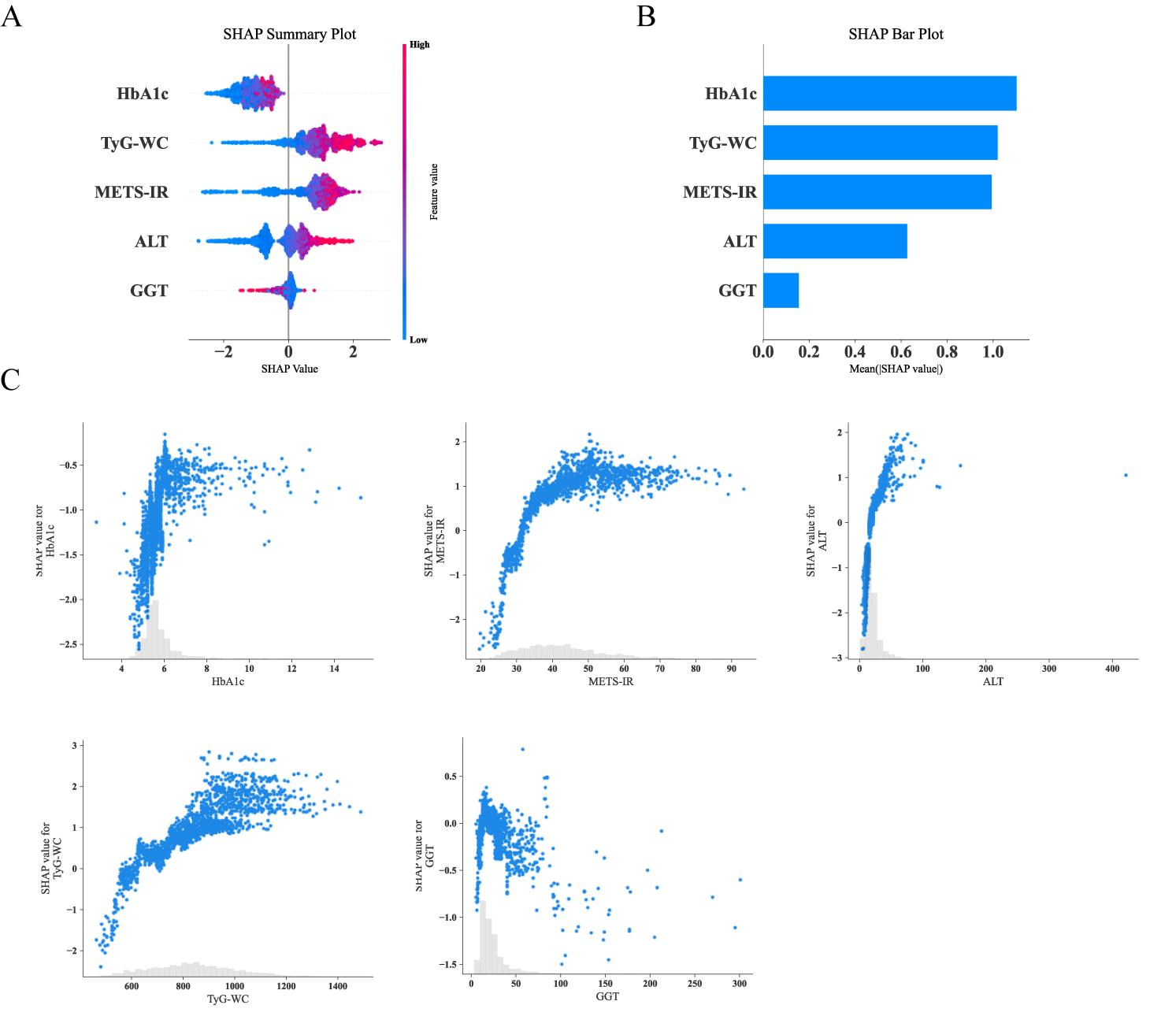


**Supplementary Table 1.** Demographic and clinical characteristics of study population in the NHANES cohort.

| Characteristic | Non-NAFLD  (N = 1,060) | NAFLD  (N = 738) | p-value |
| --- | --- | --- | --- |
| Sex |  |  | 0.019 |
| Female | 548 (52%) | 340 (46%) |  |
| Male | 512 (48%) | 398 (54%) |  |
| Age | 49 (32, 65) | 57 (42, 67) | <0.001 |
| BMI(kg/m2) | 26 (23, 29) | 31 (28, 35) | <0.001 |
| WC(cm) | 92 (82, 101) | 106 (98, 116) | <0.001 |
| Visceral fat obesity |  |  | <0.001 |
| No | 344 (32%) | 37 (5%) |  |
| Yes | 716 (68%) | 701 (95%) |  |
| Obesity |  |  | <0.001 |
| No | 450 (42%) | 58 (8%) |  |
| Yes | 610 (58%) | 680 (92%) |  |
| ALT(U/L) | 15 (11, 21) | 20 (15, 28) | <0.001 |
| AST(U/L) | 18 (15, 22) | 19 (16, 24) | <0.001 |
| GGT(U/L) | 17 (13, 24) | 24 (17, 33) | <0.001 |
| HDL(mmol/L) | 1.41 (1.19, 1.66) | 1.19 (1.01, 1.40) | <0.001 |
| TC(mmol/L) | 4.60 (3.96, 5.30) | 4.65 (4.09, 5.53) | 0.018 |
| TG(mmol/L) | 0.81 (0.59, 1.20) | 1.30 (0.89, 1.84) | <0.001 |
| TyG | 8.20 (7.85, 8.62) | 8.77 (8.38, 9.23) | <0.001 |
| TyG–BMI | 215 (183, 248) | 275 (242, 318) | <0.001 |
| TyG–WC | 758 (659, 855) | 940 (847, 1,047) | <0.001 |
| TG/HDL | 1.34 (0.86, 2.09) | 2.55 (1.54, 3.98) | <0.001 |
| METS-IR | 37 (31, 43) | 48 (42, 57) | <0.001 |
| HbA1c(%) | 5.50 (5.20, 5.80) | 5.80 (5.40, 6.20) | <0.001 |
| FPG(mmol/L) | 5.55 (5.22, 5.94) | 6.05 (5.61, 6.94) | <0.001 |
| SBP(mmHg) | 118 (108, 132) | 125 (114, 137) | <0.001 |
| DBP(mmHg) | 72 (65, 79) | 75 (68, 83) | <0.001 |

BMI, body mass index; WC, waist circumference; ALT, alanine aminotransferase; AST, aspartate aminotransferase; GGT, gamma-glutamyl transpeptidase; HDL, high-density lipoprotein; TC, total cholesterol; TG, triglycerides; FPG, fasting plasma glucose; SBP, systolic blood pressure; DBP, diastolic blood pressure.

**Supplementary Table 2.** Comparative performance metrics of NAFLD diagnostic models.

| Model | Sensitivity | Specificity | AUC |
| --- | --- | --- | --- |
| Our model | 0.929 | 0.611 | 0.902 |
| FLI^(50)^ | 0.707 | 0.581 | 0.701 |
| NAFLD-LFS^(50)^ | 0.709 | 0.958 | 0.871 |
| ION^(50)^ | 0.710 | 0.945 | 0.894 |

FLI, fatty liver index; NAFLD-LFS, NAFLD-liver fat score; ION, index of non-alcoholic steatohepatitis

50. Kouvari M, Valenzuela-Vallejo L, Guatibonza-Garcia V, Polyzos SA, Deng Y, Kokkorakis M, et al. Liver Biopsy-Based Validation, Confirmation and Comparison of the Diagnostic Performance of Established and Novel Non-Invasive Steatotic Liver Disease Indexes: Results from a Large Multi-Center Study. Metabolism (2023) 147:155666. Epub 2023/08/02. doi: 10.1016/j.metabol.2023.155666.
